# Human Trafficking ICD-10 Code Utilization in Pediatric Tertiary Care Centers Within the United States

**DOI:** 10.3389/fped.2022.818043

**Published:** 2022-02-18

**Authors:** Anjali Garg, Preeti Panda, Sindhoosha Malay, Katherine N. Slain

**Affiliations:** ^1^Department of Anesthesia and Critical Care Medicine, Johns Hopkins Children's Center, Baltimore, MD, United States; ^2^Department of Pediatric Emergency Medicine, Stanford University School of Medicine, Palo Alto, CA, United States; ^3^Case Western Reserve University School of Medicine, Cleveland, OH, United States; ^4^Department of Pediatrics, University Hospitals Rainbow Babies & Children's Hospital, Cleveland, OH, United States

**Keywords:** public health, pediatrics, child abuse, *International Classification of Diseases*, human trafficking

## Abstract

**Background:**

Human trafficking is a global public health issue that affects pediatric patients widely. The International Labor Organization estimates children comprise approximately 25% of the identified trafficked persons globally, with domestic estimates including over 2000 children a year. Trafficked children experience a broad range of health consequences leading to interface with healthcare systems during their exploitation. In June 2018, *International Classification of Diseases, Tenth Revision, Clinical Modification* (ICD-10-CM) released diagnostic codes for human trafficking.

**Objective:**

To use a large, multicenter database of US pediatric hospitalizations to describe the utilization of the ICD-10-CM codes related to child trafficking, as well as the demographic and clinical characteristics of these children.

**Methods:**

This study was descriptive in nature. Encounters using data from the Pediatric Health Information System database (PHIS) with ICD-10-CM codes indicating trafficking from June 1, 2018 to March 1st, 2020 were included in the study cohort, with data collection continuing for 30 days after first hospital encounter, until March 31st, 2020. Patients 19 years old and younger were included. Condition-specific prevalence as well as demographic and clinical characteristics for patient encounters were analyzed. Study subjects were followed for 30 days after first hospital encounter to describe healthcare utilization patterns.

**Results:**

During the study period, 0.005% (*n* = 293) of patient encounters in the PHIS database were identified as trafficked children. The children of our cohort were mostly female (90%), non-Hispanic Black (38%), and had public insurance (59%). Nearly two-thirds of patients (*n* = 190) had a documented mental health disorder at the initial encounter, with 32.1% classified as the principal diagnosis. Our cohort had a 30-day hospital inpatient, overnight observation, or emergency department readmission rate of 16% (*n* = 48).

**Discussion:**

Our study demonstrates a low utilization of human trafficking ICD-10-CM codes in academic children's health centers, with code usage predominantly assigned to Non-Hispanic Black teenage girls. As comparison, in 2019 the National Human Trafficking Hotline identified 2,582 trafficked US children in a single year. These results suggest widespread under-recognition of child trafficking in health care settings, including the intensive care unit, in addition to racial and socioeconomic disparities amongst trafficked children.

## Introduction

Human trafficking—the use of force, fraud, or coercion to perform sex or labor acts—is a devastating public health problem leading to the exploitation of children (1). The International Labor Organization estimates that children comprise approximately 25% of the identified trafficked persons globally (1–3). The prevalence and incidence of human trafficking in the United States (U.S.) is unknown, and recent estimates are based on data largely from the National Human Trafficking Hotline and the Counter Trafficking Data Collaborative (4, 5). Trafficked individuals experience a broad range of health consequences—including physical trauma, malnutrition, communicable disease, and mental health disorders—and most trafficked individuals have interfaced with the healthcare system during their exploitation (1, 6–8). Therefore, health care providers play a vital role in identifying and aiding trafficked children (1, 7–10).

In June 2018, the U.S. Centers for Disease Control and Prevention adopted diagnostic codes classifying human trafficking in the *International Classification of Diseases, Tenth Revision, Clinical Modification* (ICD-10-CM) (11, 12). The United States is the only country thus far to adopt diagnosis codes for human trafficking. From the creation of these codes, valuable data can now be collected to determine prevalence, define characteristics, and create strategies to aid children afflicted by human trafficking (10, 13). The objective of this study was to use a large, multicenter database of U.S. pediatric hospitalizations to describe the utilization of the newly adopted ICD-10-CM codes related to child human trafficking, and the demographic and clinical characteristics of these children. We hypothesized that use of these new ICD-10 codes would be low, reflecting both an under-recognition of the magnitude of this public health problem and limited awareness of the availability of coding among healthcare providers.

## Materials and Methods

### Study Design and Data Source

This was a descriptive study of the Pediatric Health Information System (PHIS), an administrative discharge database inclusive of data from 49 academic children's hospitals accounting for nearly 15% of all pediatric hospitalizations in the US (14). PHIS contains patient-level data including demographics, admission and discharge dates, and clinical and resource utilization data at the emergency department (ED), inpatient, and observation level. The database is de-identified, and encrypted patient identifiers allow for tracking of study subjects across multiple hospitalizations. Database quality is assured jointly by Children's Hospital Association (Lenexa, KS), participating hospitals, and Truven Health Analytics (Ann Arbor, MI). The study was reviewed by the University Hospitals Institutional Review Board and exempt from ethical oversight (STUDY20201822), as this was an analysis of a de-identified administrative dataset. We used STROBE cross-sectional reporting guidelines (15).

### Study Population

Children <19 years of age discharged from a PHIS hospital with ICD-10-CM codes indicating trafficking (Y076, Z62813, T7452, T7652, Z0481, Z9142, T7451, T7651, T7662, T74.62, Z04.82) from June 1, 2018 to March 1st, 2020 were included in the study cohort as index hospitalizations, with data collection continuing for 30 days after index hospitalization, until March 31st, 2020. Index hospitalization was defined as the first hospital encounter within the PHIS system during the study period. All children with an inpatient, observation, or ED level encounter at index hospitalization were included.

### Study Procedures

This was a descriptive study. We collected patient demographics, neighborhood characteristics, and clinical characteristics at index hospitalization. Demographic data included age, sex, race and ethnicity, and insurance type. Race was defined as Non-Hispanic White, Non-Hispanic Black, Hispanic or Latino, or “other,” which included Asian, Pacific Islander, Native American, and other/unknown. Insurance was categorized as private, public, or uninsured/other. Neighborhood characteristics, based on patient ZIP code, included estimated median household income and urban (dichotomized as yes or no) location. Collected clinical characteristics included presence of an infection diagnosis or mental health disorder diagnosis (both are based on a PHIS “flag”), All Patient Refined Diagnosis Related Group (APR-DRG) severity index—categorized as mild, moderate, severe, or extreme, need for admission to the pediatric intensive care unit, hospital length of stay, and hospital discharge disposition. Study subjects were followed for 30 days after index admission to describe healthcare utilization patterns, including healthcare encounter location and principle diagnoses at the repeat hospital encounter.

### Statistical Analyses

Demographics, neighborhood characteristics, and clinical characteristics of the study subjects were described using medians with interquartile range [IQR] for continuous variables and frequency with percentages for categorical variables, as appropriate. Differences based on location of index hospital encounter were described using the Wilcoxon rank-sum test or chi-square test/Fisher exact test. A *p*-value of less than 0.05 was considered as statistically significant. All analyses were conducted in SAS software, version 9.4 and R software, version 3.5.3.

## Results

During the 22-month study period, there were 6,366,691 total patients encounters in the PHIS database, of which 0.005% (*n* = 293) included an ICD-10-CM code for human trafficking. Of these 293 included encounters, 57% (*n* = 167) occurred in the ED. Frequency and descriptors of the ICD-10 codes during the study period are included in Table 1. The most common code utilized in the ED was Y07.6, “multiple perpetrators of maltreatment and neglect” (33.8%), while the most common code utilized among hospital encounters was Z62.813, “personal history of forced labor or sexual exploitation in childhood” (21.2%; Table 1). Ninety percent (*n* = 264) of included children were female (90.1%), with a median age of 15 years [IQR 13–16]. The majority (*n* = 112) identified as Non-Hispanic Black, with Non-Hispanic White identification as the second most common (*n* = 83). Nearly two-thirds of patients (*n* = 190) had a documented mental health disorder at the initial encounter, with 32.1% classified as the principal diagnosis. There were differences in insurance status, illness severity, illness characteristics, and discharge disposition between ED and hospital encounters (Table 2). Most providers utilizing human trafficking ICD-10-CM codes were emergency medicine physicians (42.7%), pediatricians (24.2%), and psychiatrists (7.8%; Table 3).

**Table 1:** Frequency of human trafficking ICD-10 codes utilized by healthcare providers during study period.

***International Classification of Diseases (ICD)−10 codes***		***N* (%) (*n* = 293)**
Y076	Multiple perpetrators of maltreatment and neglect	99 (33.8)
Z62813	Personal history of forced labor or sexual exploitation in childhood	62 (21.2)
T7452	Child sexual exploitation, confirmed	60 (20.5)
T7652	Child sexual exploitation, suspected	50 (17.1)
Z0481	Encounter for examination and observation of victim following forced sexual exploitation	15 (5.1)
Z9142	Personal history of forced labor or sexual exploitation	4 (1.4)
T7451	Adult forced sexual exploitation—confirmed	1 (0.3)
T7651	Adult forced sexual exploitation—suspected	1 (0.3)
T7662	Child forced labor exploitation, suspected	1 (0.3)
T7462	Child forced labor exploitation, confirmed	0 (0)
Z0482	Encounter for examination and observation of victim following forced labor exploitation	0 (0)

**Table 2:** Descriptive statistics of pediatric trafficking encounters based on encounter location, 2018 Q3 to 2019 Q3.

	**Overall encounters**	**ED encounters**	**Hospital encounters**	***p*-value**
	**(*n* = 293)**	**(*n* = 167)**	**(*n* = 126)**	
**ICD-10 code^a^**, ***n (%)***				<0.001
Y07.6	99 (33.8)	78 (46.7)	21 (16.7)	
Z62.813	62 (21.2)	13 (7.8)	49 (38.9)	
T74.52	60 (20.5)	39 (23.4)	21 (16.7)	
T76.52	50 (17.1)	21 (12.6)	29 (23.0)	
Z04.81	15 (5.1)	14 (8.4)	1 (0.8)	
Others	7 (2.4)	2 (1.2)	5 (4.0)	
**Age, years**	15 [13–16]	15 [13–16]	15 [14–16]	0.126
**Female**, ***n*** **(%)**	264 (90.1)	146 (87.4)	118 (70.7)	0.117
**Race/Ethnicity**^b^, ***n*** **(%)**				0.993
Non-hispanic black	112 (38.2)	65 (38.9)	47 (37.3)	
Non-hispanic white	83 (28.3)	47 (28.1)	36 (28.6)	
Hispanic or latino	67 (22.9)	38 (22.8)	29 (23.0)	
Other	20 (6.8)	11 (6.6)	9 (7.1)	
**Estimated household income^c^**, ***$US***	37,720 [29,030–48,556]	36,189 [29,293–48,699]	41,590 [30,127–49,437]	0.115
**Urban (%)^d^**, ***n*** **(%)**	258 (88.1)	148 (88.6)	110 (87.3)	0.942
**Insurance**, ***n*** **(%)**				0.002
Public	173 (59.0)	86 (51.5)	87 (69.0)	
Private	63 (21.5)	39 (23.4)	24 (19.0)	
Uninsured	25 (8.5)	22 (13.2)	3 (2.4)	
Others	32 (10.9)	20 (12.0)	12 (9.5)	
**Illness severity**, ***n*** **(%)**				<0.001
Minor	113 (38.6)	95 (56.9)	10 (7.9)	
Moderate	116 (44.1)	56 (33.5)	46 (36.5)	
Major	56 (19.1)	14 (8.4)	30 (23.8)	
Extreme	7 (2.4)	1 (0.6)	2 (1.6)	
**Infection diagnosis**, ***n (%)***	63 (21.5)	16 (9.6)	47 (37.3)	<0.001
**Mental health disorder diagnosis**, ***n*** **(%)**	190 (64.8)	76 (45.5)	114 (90.5)	<0.001
**Discharge disposition^e^**, ***n*** **(%)**				<0.001
Routine	218 (74.4)	148 (88.6)	70 (55.6)	
Inpatient psychiatric unit	35 (11.9)	10 (6.0)	25 (19.8)	
Another health care facility	30 (10.2)	5 (3.0)	25 (19.8)	
Law enforcement	4 (1.4)	3 (1.8)	1 (0.8)	
Died	1 (0.3)	0 (0.0)	1 (0.8)	
**30-day repeat patient encounter**, ***n*** **(%)**	55 (18.7)	18 (10.7)	30 (23.8)	0.077

*Q, quarter; ED, emergency department; ICD-10, International Classification of Diseases, Tenth Revision; US, United States; data presented as n (%) or median [interquartile range], as appropriate*.

*^#a^ICD-10 codes include: Y07.6, multiple perpetrators of maltreatment or neglect; Z62.813, personal history of forced labor of sexual exploitation in childhood; T74.52, child sexual exploitation—confirmed; T76.52, child sexual exploitation—suspected; Z04.81, encounter for examination and observation of victim following forced sexual exploitation; “other” includes T74.51, adult forced sexual exploitation—confirmed (n = 1); T76.51, adult forced sexual exploitation—suspected (n = 1); T76.62, child forced labor exploitation, suspected (n = 1); Z91.42, personal history of forced labor or sexual exploitation (n = 4)*;

b *8 encounters with missing race data*;

c *11 encounters with missing income data*;

d *11 encounters with missing urban data*;

e *5 patients with unknown disposition data*.

**Table 3:** Physician subspecialty and utilization of ICD-10 codes.

	***N* (%) (*n* =293)**
**Pediatric sub-specialties**
Emergency medicine	125 (42.7)
General pediatrics	71 (24.2)
Psychiatry	23 (7.8)
Hospital medicine	13 (4.4)
Adolescent medicine	5 (1.7)
Surgery–pediatric/general	3 (1.0)
Critical care medicine	2 (0.7)
Endocrinology	2 (0.7)
Infectious disease	2 (0.7)
Sports medicine	2 (0.7)
Child Abuse	1 (0.3)
**Other sub-specialties**
Obstetrics-gynecology	3 (1.0)
Not otherwise specified	33 (11.9)
Anesthesiology	3 (1.0)

Nineteen percent (*n* = 55) of children had a healthcare visit at a PHIS hospital within 30 days of the initial encounter, with a 30-day hospital inpatient/observation or ED readmission rate of 16% (*n* = 48; Table 2). One-third of principal diagnoses at the 30-day re-encounter were categorized as child maltreatment including ICD-10 codes for sexual abuse, physical abuse and trafficking/exploitation (32.7%), followed by codes for mental health disorders (30.9%), and physical trauma (14.5%; Table 4).

**Table 4:** Principal ICD-10 diagnoses for healthcare visits within 30 days of initial encounter^a^.

**Principal ICD-10 diagnosis codes**	**Frequency (%) (*N* = 55)**
**Child abuse or trafficking (exploitation)** → T74.22—child sexual abuse confirmed initial → T76.52—child sexual exploitation suspected initial → T74.52—child sexual exploitation confirmed initial → Z04.81—encounter for exam & observation of victim with forced sex exploitation → T74.12—child physical abuse confirmed initial → T76.12—child physical abuse suspected initial → T76.22—child sexual abuse suspected initial → T76.52—child sexual exploitation suspected sequela	18 (32.7)
**Mental health disorders** → R45.851—suicidal ideations → T43.222—poisoning by anti-depressant, intentional self-harm initial → T43.632—poison methylphenidate intentional self-harm initial → T46.5X2—poison by anti-hypertension drugs for self-harm initial → T50.902—poison drug, substance not specified self-harm initial → F31.2—bipolar disorder current episode manic severe with psych features → F32.89—depressive episodes → F33.2—major depressive disorder recurrent severe without psychotic features → F43.10—post-traumatic stress disorder → F32.9—major depressive disorder single episode	17 (30.9)
**Physical trauma (excluding abuse)** → S09.90X—injury head, not specified → S02.2XX—fracture nasal bones initial closed fracture → S02.602—open fracture of left mandible → S06.2X9—diffuse traumatic brain injury with loss of consciousness → S06.5X9—traumatic subdural hemorrhage with loss of consciousness → S09.93X—injury face, not specified → R41.89—symptoms and signs involving cognitive function and awareness	8 (14.5)
**Reproductive health** → A56.8—sexually transmitted chlamydial infect sites → N89.8 - non-inflammatory disorders vagina → O42.913—preterm premature rupture of membranes onset labor	3 (5.5)
**Miscellaneous** → B33.8—viral diseases → E10.10—type 1 diabetes mellitus with ketoacidosis, but without coma → E11.65—type 2 diabetes mellitus with hyperglycemia → G40.909—not specified epilepsy without status epilepticus → J81.1—chronic pulmonary edema → K80.20—calculus gallbladder without cholecystitis or obstruction → L02.414—cutaneous abscess left upper limb → R04.0—epistaxis → T18.9XX—foreign body alimentary tract, not specified	9 (16.4)

*ICD-10, International Classification of Diseases, Tenth Revision; data presented as n (%) as appropriate*.

a *Seven of the 55 readmission encounters were clinic visits, 30 were inpatient or observation encounters, and 18 were emergency department encounters*.

## Discussion

Using a large multicenter dataset, we described the prevalence, demographics, and clinical characteristics of trafficked children seeking healthcare at 49 US academic children's hospitals based on the utilization of newly adopted ICD-10-CM codes. ICD-10-CM codes were used as a surrogate in this study to designate awareness of human trafficking amongst pediatric patients. We had several important findings. First, we demonstrated a low use of trafficking ICD-10-CM codes among children receiving care in the ED or admitted to the hospital. Second, mental health disorders were common in this cohort, and third, most identified trafficked children were Non-Hispanic Black adolescent girls from urban neighborhoods primarily coded for sex trafficking. Our findings suggest there is an urgent need to educate healthcare providers on the importance of screening and identifying trafficked children.

During the 22-month study period, only 0.005% of patient encounters in the PHIS database were identified as trafficked children. In 2019, the National Human Trafficking Hotline identified 2,582 trafficked U.S. children (4). The true prevalence of trafficking among U.S. children is unknown. Multiple organizations, including the U.S. Department of State, acknowledge current estimates of human trafficking are inaccurate because it is an elusive crime and identifying victims is challenging (16–18). Similar to previous studies showing underuse of ICD codes among physically abused children, the utilization of ICD-10-CM trafficking codes in our study likely underrepresents the actual number of trafficked children in the PHIS database (19). Healthcare providers are more likely to be aware that trafficking exists, but not equipped to recognize signs of trafficking (20, 21). Additionally, provider perception of trafficked individuals could bias who is being screened for trafficking (21). Our study found higher use of ICD-10-CM codes among pediatric ED and psychiatry subspecialty providers. This likely reflects an increased awareness related to the known association between trafficking and acute exacerbations of underlying psychiatric conditions requiring immediate medical attention (6, 7, 22). Nevertheless, trafficked individuals may present to any clinical setting (23). A recent case series of trafficked pediatric patients admitted to the intensive care unit concluded the severity of their illness was potentially exacerbated due to poor access to medical care (23). Provider bias and the elusive nature of human trafficking emphasize that a high index of suspicion should be maintained for identification of trafficking, indicating the need for universal education and training (7, 9).

Confidentiality concerns for trafficked children likely contributes to low documentation and low diagnosis code utilization. ICD-10-CM diagnoses codes may be viewable on printed discharge materials, electronic patient portals, insurance summaries, and in the electronic health record (10, 11). Safety concerns that may discourage the use of these codes include the risk of health information being viewed by the patient's trafficker, potential legal implications such as in immigration proceedings, and a risk of discrimination toward trafficked persons (10, 11). Because of the importance of reliable documentation, hospitals caring for at-risk children should preemptively develop confidential systems to safely identify these patients (11).

Trafficked children may also be reluctant to seek healthcare. In a systematic review, three major sub-groups of themes were identified and noted to be significant barriers to healthcare utilization for trafficked children (24). Some of the barriers included trafficker control, physical confinement, lack of trust in healthcare providers, concerns about confidentiality, decreased knowledge of the healthcare system, and emotional reluctance (24). Cost and long appointment wait times were also cited (24). Another systematic review also reported legal repercussions, perception of biased care, trafficker control, or lack of interest in engagement with healthcare for various reasons facilitated as barriers to seeking healthcare (25). These barriers indicate that a decrease in ICD-10 codes utilization could also be due to decreased presentation of trafficked children to healthcare systems. The provision of trauma informed, patient centered care could reduce many of these survivor barriers to seeking healthcare (24, 26).

Psychiatric illness was common in our cohort of children. Nearly two-thirds received a mental health disorder diagnosis at the initial hospital visit, and nearly one-third of the principal diagnoses were mental health-related among those children seen within 30 days of the index hospitalization. Our data is consistent with prior literature demonstrating a high prevalence of psychiatric disease among trafficked youth both domestically and internationally (6, 8, 22, 27, 28). Two U.S. based studies found high rates of mental health disorders, including posttraumatic stress disorder, depression, and substance abuse amongst trafficked youth (8, 28). Internationally, a study of 207 trafficked women and adolescent girls in Europe reported 56% of the participants had symptoms consistent with posttraumatic stress disorder and depression (6, 27). Similar results were seen in Southeast Asia's Mekong region, with high rates of depression and suicide amongst trafficked teenage girls (27). Increase in the provision of mental health support and trauma informed care can reduce the impact of psychiatric illness in this vulnerable population.

Trafficked children are at high risk of re-exploitation, especially by family members (5). Most patients in our study were discharged home leading to potential re-exploitation. Of the study participants, 19% sought medical care within 30 days of the initial encounter with abuse-related, trafficking-related, and mental health-related principal diagnoses being the most common. The readmission diagnoses emphasize the cycle of re-exploitation, indicating that further work is necessary to reduce re-traumatization and develop safe discharge plans for these children.

Most children in our cohort were Non-Hispanic Black teenage girls living in urban, low-income neighborhoods. While human trafficking affects children across race and socioeconomic lines, existing literature and data reports have suggested that human trafficking disproportionately affects racial and ethnic minorities, consistent with our results (28–31). A single center retrospective study at a pediatric hospital of 63 patients reported that 98% of their participants identified as female and 54% identified as Non-Hispanic Black (30). Another multi-center study of 84 all female patients reported 56% as Non-Hispanic Black (28). However, there are few studies from larger national cohorts that comprehensively examine the racial and ethnic profile of trafficked youth, and the disparities associated (31). While, our study draws from a national sample, it is still most likely an underestimation of child human trafficking for the aforementioned reasons, demonstrating a research gap in the field requiring further exploration of sociodemographic characteristics of trafficked youth.

Identified codes in our study were utilized almost exclusively for sexual exploitation, suggesting limited awareness of labor trafficking. In one year, the National Human Trafficking Hotline identified over 1,300 cases of labor trafficking, including 115 cases of child labor, a number that is again likely a vast underestimation of the problem, but still higher than the one labor exploitation code utilized in this study cohort (4, 32).

This study has limitations. First, this study included children's hospitals that participate in PHIS, a database limited to tertiary and quaternary care pediatric hospitals, affecting the generalizability of our results; ICD-10-CM code utilization for trafficked children evaluated at smaller, rural, or non-academic centers is not described by this study. Second, the human trafficking ICD-10-CM codes have only recently become available, and there may be a natural increase in code usage as awareness of their availability increases amongst providers. Lastly, the codes could be underutilized due to the provider hesitancies described above, including concerns for patient safety.

## Conclusion

This large multicenter descriptive study demonstrated a low utilization of human trafficking ICD-10-CM codes by health care providers in academic children's health centers, and the trafficking codes that were used were predominantly assigned to Non-Hispanic Black teenage girls. While prospective studies are necessary to validate our findings, these results suggest there is widespread under-recognition of child trafficking in health care settings, in addition to racial and socioeconomic disparities amongst trafficked children. Until awareness improves children will go unaided, further exacerbating an already devastating public health problem. Health care institutions should act immediately to systematize provider education, patient screening and identification, and implement safe, accessible, trauma-informed interventions for trafficked children.

## Data Availability Statement

The original contributions presented in the study are included in the article, further inquiries can be directed to the corresponding author.

## Author Contributions

AG and PP conceptualized the manuscript, conducted a literature search, and drafted the initial manuscript. SM conducted statistical analysis on the data. KS conceptualized the manuscript, provided guidance on data analysis, and reviewed and revised the manuscript. All authors approved the final manuscript and agree to be accountable for all aspects of the work.

## Conflict of Interest

The authors declare that the research was conducted in the absence of any commercial or financial relationships that could be construed as a potential conflict of interest.

## Publisher's Note

All claims expressed in this article are solely those of the authors and do not necessarily represent those of their affiliated organizations, or those of the publisher, the editors and the reviewers. Any product that may be evaluated in this article, or claim that may be made by its manufacturer, is not guaranteed or endorsed by the publisher.

## Abbreviations

US: United States
ICD-10-CM: *International Classification of Diseases, Tenth Revision, Clinical Modification*
PHIS: Pediatric Health Information System
ED: emergency department
APR-DRG: All Patient Refined Diagnosis Related Group
IQR: interquartile range
Q: Quarter.
